# Blood Serum From Head and Neck Squamous Cell Carcinoma Patients Induces Altered MicroRNA and Target Gene Expression Profile in Treated Cells

**DOI:** 10.3389/fonc.2018.00217

**Published:** 2018-06-11

**Authors:** Brittany Allen, Augusto Schneider, Berta Victoria, Yury O. Nunez Lopez, Mark Muller, Mateusz Szewczyk, Jakub Pazdrowski, Ewa Majchrzak, Wojciech Barczak, Wojciech Golusinski, Pawel Golusinski, Michal M. Masternak

**Affiliations:** ^1^Burnett School of Biomedical Sciences, College of Medicine, University of Central Florida, Orlando, FL, United States; ^2^Faculdade de Nutrição, Universidade Federal de Pelotas, Pelotas, Brazil; ^3^Translational Research Institute for Metabolism and Diabetes, Florida Hospital, Orlando, FL, United States; ^4^Epigenetics Division, TopoGEN Inc, Buena Vista, CO, United States; ^5^Department of Head and Neck Surgery, Poznań University of Medical Sciences, The Greater Poland Cancer Centre, Poznan, Poland; ^6^Biology and Environmental Studies, Head and Neck Cancer Biology Laboratory, Poznań University of Medical Sciences, Poznan, Poland

**Keywords:** head and neck squamous cell carcinomas, microRNA, sequencing data analysis, p53 pathway, cancer-associated factors

## Abstract

The head and neck squamous cell carcinoma (HNSCC) represents one of the most common cancers in humans. Close to 600,000 new diagnoses are made every year worldwide and over half of diagnosed patients will not survive. In view of this low survival rate, the development of novel cell-based assays for HNSCC will allow more mechanistic approaches for specific diagnostics for each individual patient. The cell-based assays will provide more informative data predicting cellular processes in treated patient, which in effect would improve patient follow up. More importantly, it will increase the specificity and effectiveness of therapeutic approaches. In this study, we investigated the role of serum from HNSCC patients on the regulation of microRNA (miRNA) expression in exposed cells *in vitro*. Next-generation sequencing of miRNA revealed that serum from HNSCC patients induced a different miRNA expression profile than the serum from healthy individuals. Out of 377 miRNA detected, we found that 16 miRNAs were differentially expressed when comparing cells exposed to serum from HNSCC or healthy individuals. The analysis of gene ontologies and pathway analysis revealed that these miRNA target genes were involved in biological cancer-related processes, including cell cycle and apoptosis. The real-time PCR analysis revealed that serum from HNSCC patients downregulate the expression level of five genes involved in carcinogenesis and two of these genes—P53 and SLC2A1—are direct targets of detected miRNAs. These novel findings provide new insight into how cancer-associated factors in circulation regulate the expression of genes and regulatory elements in distal cells in favor of tumorigenesis. This has the potential for new therapeutic approaches and more specific diagnostics with tumor-specific cell lines or single-cell *in vitro* assays for personalized treatment and early detection of primary tumors or metastasis.

## Introduction

Head and neck squamous cell carcinomas (HNSCC) are squamous cell cancers originating in the lip and the oral cavity, oropharynx, hypopharynx, or larynx. The etiologies of HNSCC are divided into two groups corresponding to the two major risk factors for this type of cancer: (1) exposure to alcohol and tobacco and (2) infection with human papillomavirus (HPV).

Smoking increases the risk of HNSCC 5- to 25-fold (1) and the risk is even higher when habitual smoking and alcohol use are combined. Over the past decade, there has been a decrease in smoking- related HNSCC’s cases. This is correlated with a decrease in the use of tobacco products. This decline is occurring congruently with an increase in HPV-positive HNSCC incidences, particularly in younger individuals (1–4).

MicroRNAs (miRNAs) are small, non-coding RNA molecules that are involved in gene regulation. After being transcribed, primary miRNA transcripts are processed into pre-miRNA hairpin structures before being cleaved into short, dsRNA fragments. Finally, one strand of the fragment is then degraded to form the mature miRNA (5). MiRNAs participate in the regulation of gene expression by forming RNA-induced silencing complexes that target complementary sequences on mRNA and either inhibit translation or cause degradation (6). Further, miRNAs can contribute to tumorigenesis either by upregulation of a miRNA targeting tumor suppressor genes or downregulation of a miRNA targeting oncogenes (7). For example, miR124a is frequently downregulated in several cancer types, including colon, breast, and lung carcinomas, as well as some leukemias and lymphomas. Because this miRNA is a negative regulator of CDK6, downregulation caused by hypermethylation results in increased levels of CDK6, which in turn facilitates inactivation of RB1 *via* phosphorylation (8). miRNAs have also been shown to regulate epigenetic processes by targeting DNMTs and histone methylating EZH2 complexes (9–11). Aberrant miRNA expression can result from chromosomal deletions, gene mutations involved in miRNA processing, or by epigenetic mechanisms that affect miRNA expression (12). DNA methylation and chromatin remodeling processes can cause dysregulation of miRNA in the same way as in gene encoding transcripts (13–15). This is evidenced by the ability of HDAC inhibitors (16, 17) and hypomethylating drugs (18) to induce changes in miRNA expression, suggesting interdependent regulation between these mechanisms.

Since serum samples are easily accessible in a clinical setting, they are often examined for the presence of biomarkers associated with various disease states. Studies have demonstrated that specific miRNA expression profiles can be identified between cancer tissue and adjacent healthy tissue in HNSCC patients (19). In our previous study, we also showed different expression levels between serum from HNSCC patients and serum from healthy individuals (20). There are also studies indicating alterations in miRNA levels when comparing serum from HNSCC patients before and after treatment (21). Further, several of these miRNAs have been shown to have diagnostic or prognostic value (22).

In vitro studies have demonstrated that serum from cancer patients generate tumorigenic phenotypes in cultured cells (23–25). This can occur by horizontal gene transfer from circulating, cell-free DNA (25, 26), or by the uptake of extracellular vesicles that are released into circulation by cancer cells (27–30). Extracellular vesicles, or exosomes, are membrane bound vesicles that may contain membrane or cytosolic proteins, lipids, or nucleic acids with roles in intercellular signaling (31). Released by both healthy and cancer cells, exosomes are found in many different body fluids, including urine, breast milk, blood, amniotic fluid, ascites, semen, and saliva (27, 31). Cancer cells use this mechanism to package and deliver oncogenic proteins (32, 33), mRNA, miRNA (32, 34, 35), and DNA (28) This mechanism is capable of causing the malignant transformation of recipient cells, or it can facilitate cancer progression and metastasis (27).

In this study, we investigated the potential of serum from HNSCC patients to affect the regulation of miRNA expression in exposed cells, which could provide novel approaches in future diagnostic *in vitro* studies using a variety of well established and genetically characterized cell lines for HNSCC, as well as other types of cancer.

## Materials and Methods

### Human Blood Serum Collection

Serum was collected and pre-processed in the Department of Head and Neck Surgery, Greater Poland Cancer Center before surgical treatment (Table 1). The Institutional Review Board of the University of Medical Sciences in Poznan approved the study, and both informed and written consents were obtained from all patients. Blood samples were collected in BD Vacutainer Serum Separation Tubes, incubated for 15 min at room temperature to allow coagulation, and centrifuged at 1,300 *g* for 10 min. The serum supernatant was transferred to new tubes, centrifuged at 16,000 *g* for 15 min to remove any residual cells and debris, and stored at −80°C (20).

**Table 1:** Head and neck squamous cell carcinomas (HNSCC) patients and healthy control info.

HNSCC patients	Age	TNM	Tumor grade	Tumor localization
1	60	T3N2M0	G2	Oropharynx
2	58	T2N1M0	G2	Oral
3	55	T4N2M0	G2	Larnyx
4	50	T4N2M0	G2	Larnyx
5	61	T2N3M0	G3	Oral/oropharnyx
6	59	T2N0M0	G1	Oral

### Cell Culture

HeLa cells were grown in RPMI basal medium with l-glutamine and supplemented with 10% FBS and 1% penicillin–streptomycin. Cells were grown in an incubator at 37°C at 5% CO_2_.

### HNSCC Serum Treatments

HeLa cells were plated in 24-well plate (Corning) at low density and allowed to attach for several hours. Medium was then removed and replaced with freshly prepared medium containing 10% human serum and no FBS. A total of 11 serum samples were used, 7 from HNSCC patients and 4 from healthy individuals. Cells were allowed to grow for 72 h before extracting RNA.

### MiRNA Extraction

Total RNA was extracted from a confluent well of a 24-well plate of serum-treated cells (*n* = 11) using RNeasy Universal Plus Mini Kit (Qiagen) according to the manufacturer’s instructions. RNA concentration and purity was determined using a plate reader (Epoch™ Mircoplate Spectrophotometer, BioTek, Winooski, VT, USA).

### MiRNA Library Preparation and Sequencing

Libraries for miRNA sequencing were prepared from RNA from serum and serum-treated cells using NEBNEXT Small RNA Library Prep Set for Illumina (New England Biolabs). Libraries were prepared according to the manufacturer’s protocol, and each sample was given a unique index primer (a barcode). After that, libraries were size selected using a 6% polyacrylamide gel. The quantity and quality of miRNA libraries was determined using a BioAnalyzer and RNA Nano Lab Chip Kit (Agilent Technologies, Santa Clara, CA, USA). The barcoded samples were equimolarly combined in a single microcentrifuge tube and submitted to sequencing on a HiSeq 2500 instrument (Illumina Inc.). RNA-Seq data are available at the sequence read archive at NCBI under accession number SRP144712.

Sequencing data were analyzed using a sRNA toolbox (36) for alignment and quantification of miRNA libraries and EdgeR (37) was used for statistical analyses of differentially expressed miRNAs. MiRNAs with a FC > 1.3 and FDR < 0.05 were considered to be upregulated; and those with FC < 0.70 and FDR < 0.05 were considered to be downregulated. Unsupervised hierarchical clustering was performed using the bioconductor package DESeq (1.2.0) and data for the 50 most expressed miRNAs was collected (38). MiRNA families were identified using miRBase (39). Analyses using R packages were implemented in the R (3.2.2) programming environment.

### Target and Pathway Analysis of miRNA-seq

DIANA-mirPath (v.3) was used for gene ontology (GO) analysis of biological processes and KEGG molecular pathways (40, 41) using validated gene interactions of the differentially regulated miRNAs identified in Tarbase v7.0 database (42). *P* values lower than 0.05 were considered significant for pathway and GO terms enrichment analyses. Only KEGG pathways with at least nine targeted genes targeted by at least six miRNAs were reported.

### Real-Time PCR

Cells were seeded in 24-well plate in normal culture medium with FBS. After cells attached, media was removed and replaced with freshly prepared media supplemented with 10% human serum in place of FBS and allowed to grow for 72 h. Cells were lifted and RNA was extracted using RNeasy Plus Universal Mini Kit (Qiagen) according to kit manual. RNA concentration and purity was determined using a plate reader (Epoch™ Mircoplate Spectrophotometer, BioTek, Winooski, VT, USA).

RNA was converted to cDNA using iScript cDNA synthesis kit (Bio-Rad) according to the manufacturer protocol using 40 µL reaction volumes. The cDNA was then diluted to 80 µL. Reactions were set up in a MicroAmp^®^ Fast Optical 96-well reaction plate (Applied Biosystems) with 2 µL of diluted cDNA, 0.2 µL each of forward and reverse primer, 12.6 µL of nuclease free water, and 5 µL of Fast SYBR Green Master Mix (Applied Biosystems) per well. Quantitative real-time PCR (qPCR) was performed using a 7900 HT Fast system (Applied Biosystems) at 95°C for 20 s, followed by 45 cycles of 1 s denaturation at 95°C and 20 s annealing/extension at 62°C. Primer sequences are listed in Table 2. Beta-2-microglobulin (B2M) was used as the housekeeping gene to normalize qPCR data. Relative gene expression in each sample was calculated using the following equation: 2^A–B^/2^C–D^ [A = Ct value of the gene of interest in the first control sample (cell line treated with serum from healthy human), B = Ct value of the gene of interest in each sample, C = Ct value of B2M in the first control sample (cell line treated with serum from healthy human), and D = Ct value of B2M in each sample]. This gives the first control sample a relative expression of 1 and all other samples are calculated in relation to this sample. The results of the cells treated with serum from healthy individuals (control group) were averaged and results of all samples were divided by this average to express the fold change in expression of the genes of interest in relation to the average of the control group (cells treated with healthy human serum) (43).

**Table 2:** List of primers used to determine relative gene expression.

Gene	Primer	Sequence
Beta-2-microglobulin (B2M)	F′	5′-GAGTATGCCTGCCGTGTGAA-3′
B2M	R′	5′-CGGCATCTTCAAACCTCCAT-3′
TP53	F′	5′-GTGCAGCTGTGGGTTGATTC-3′
TP53	R′	5′-GCCAGACCATCGCTATCTGA-3′
RB1	F′	5′-TCAGAAGGTCTGCCAACACC-3′
RB1	R′	5′-CAGAAGTCCCGAATGATTCACC-3′
CDC20	F′	5′-AATGCGCCAGAGGGTTATCA-3′
CDC20	R′	5′-CGGCCAGTACATTCCCAGAA-3′
SLC2A1	F′	5′-GAACTCTTCAGCCAGGGTCC-3′
SLC2A1	R′	5′-ACCACACAGTTGCTCCACAT-3′
DNMT1	F′	5′-GATCGAGACCACGGTTCCTC-3′
DNMT1	R′	5′-CGGCCTCGTCATAACTCTCC-3′
DNMT3A	F′	5′-GGGGGAGGCACTTGACAC-3′
DNMT3A	R′	5′-CTCTGTCAGCCTGTGGGTG-3′
DNMT3B	F′	5′-ATAAGTCGAAGGTGCGTCGT-3′
DNMT3B	R′	5′-TGTGCGTCTTCGAGTCTTGT-3′
CDKN2A	F′	5′-TGCCCAACGCACCGAAT-3′
CDKN2A	R′	5′-CGGGTGAGAGTGGCGG-3′
SMARCA4	F′	5′-CGCAAGGAGGTGGACTACAG-3′
SMARCA4	R′	5′-AGCGTGCCCTCCTCGAT-3′
CCND1	F′	5′-GCCGAGAAGCTGTGCATC-3′
CCND1	R′	5′-GGCCAGGTTCCACTTGAG-3′
GFP	F′	5′-GCTCGATGCGGTTCACCAG-3′
GFP	R′	5′-GCTCGATGCGGTTCACCAG-3′
HRAS	F′	5′-GGACGAATACGACCCCACTAT-3′
HRAS	R′	5′-TGTCCAACAGGCACGTCTC-3′
NOTCH1	F′	5′-AGCCTCAACGGGTACAAGTG-3′
NOTHC1	R′	5′-GCCACTGGTCATGTCTTTGC-3′
MDM2	F′	5′-AGGAGATTTGTTTGGCGTGC-3′
MDM2	R′	5′-TGAGTCCGATGATTCCTGCTG-3′
PTEN	F′	5′-ACTTGCAATCCTCAGTTTGTGG-3′
PTEN	R′	5′-AACTTGTCTTCCCGTCGTGT-3′

### Statistical Analysis of Relative Gene Expression

Relative expression of genes of interest for cells treated with serum from healthy or cancer patients were subjected to a two-tailed *t*-test with a 95% confidence interval and graphed with mean ± SEM using Prism 5 software (GraphPad).

## Results

### HNSCC Patient Serum Alters the miRNA Profile of Treated Cells

In order to investigate the effects of cancer-associated circulating factors on cells in culture, we treated HeLa cells with serum from HNSCC patients and healthy donors. Next-generation sequencing of miRNA revealed that serum from HNSCC patients induced a different miRNA expression profile than the serum from healthy individuals. We detected a total of 377 miRNA expressed in HeLa cells and found a total of 16 miRNAs that were differentially expressed (Table 3): 12 were downregulated and 4 were upregulated.

**Table 3:** List of microRNA (miRNA) differentially expressed between cells treated with head and neck squamous cell carcinomas (HNSCC) patient serum and with normal human serum.

miRNA	Healthy^a^^,^^b^	Cancer^a^^,^^c^	FC^d^	*P*-value	False discovery rate
**Downregulated**					

hsa-miR-216b-5p^e^	1,945.6 ± 753.8	821.9 ± 204.0	0.42	0.0000	0.0000
hsa-miR-128-3p^e^	6,633.9 ± 762.8	4,384.1 ± 636.8	0.66	0.0001	0.0049
hsa-miR-216a-3p	317.0 ± 130.3	165.1 ± 20.5	0.52	0.0001	0.0049
hsa-miR-4443	50.9 ± 25.6	16.6 ± 12.1	0.33	0.0003	0.0206
hsa-miR-24-1-5p	38.7 ± 8.6	20.7 ± 4.9	0.53	0.0005	0.0223
hsa-miR-212-5p	225.6 ± 44.9	142.5 ± 18.4	0.63	0.0005	0.0225
hsa-miR-424-3p	169.9 ± 51.3	104.1 ± 13.0	0.61	0.0010	0.0330
hsa-miR-4483^e^	38.9 ± 13.1	19.5 ± 6.0	0.50	0.0009	0.0330
hsa-miR-132-5p	557.4 ± 61.2	312.6 ± 144.3	0.56	0.0012	0.0353
hsa-miR-216a-5p^e^	169.8 ± 69.6	88.1 ± 34.3	0.52	0.0016	0.0369
hsa-miR-32-5p	607.1 ± 71.7	392.8 ± 111.7	0.65	0.0015	0.0369
hsa-miR-5100	400.9 ± 74.8	264.2 ± 53.6	0.66	0.0021	0.0465

**Upregulated**					

hsa-miR-31-3p	110.6 ± 13.8	246.9 ± 92.1	2.23	0.0000	0.0018
hsa-miR-143-5p	1.1 ± 1.1	7.9 ± 3.0	5.81	0.0000	0.0025
hsa-miR-30c-2-3p	709.6 ± 51.0	981.4 ± 64.2	1.38	0.0011	0.0348
hsa-miR-135b-5p	4.5 ± 2.6	13.5 ± 4.2	3.03	0.0005	0.0223

*^a^miRNA reads per million*.

*^b^Cells treated with healthy human serum, n = 4*.

*^c^Cells treated with HNSCC patient serum, n = 7*.

*^d^Fold change in cancer serum compared with normal human serum*.

*^e^Several differentially expressed miRNAs were not found in Tarbase, so they were excluded from pathway analysis*.

### Key Pathways Are Targeted by Differentially Expressed miRNAs

**Figure 1:**
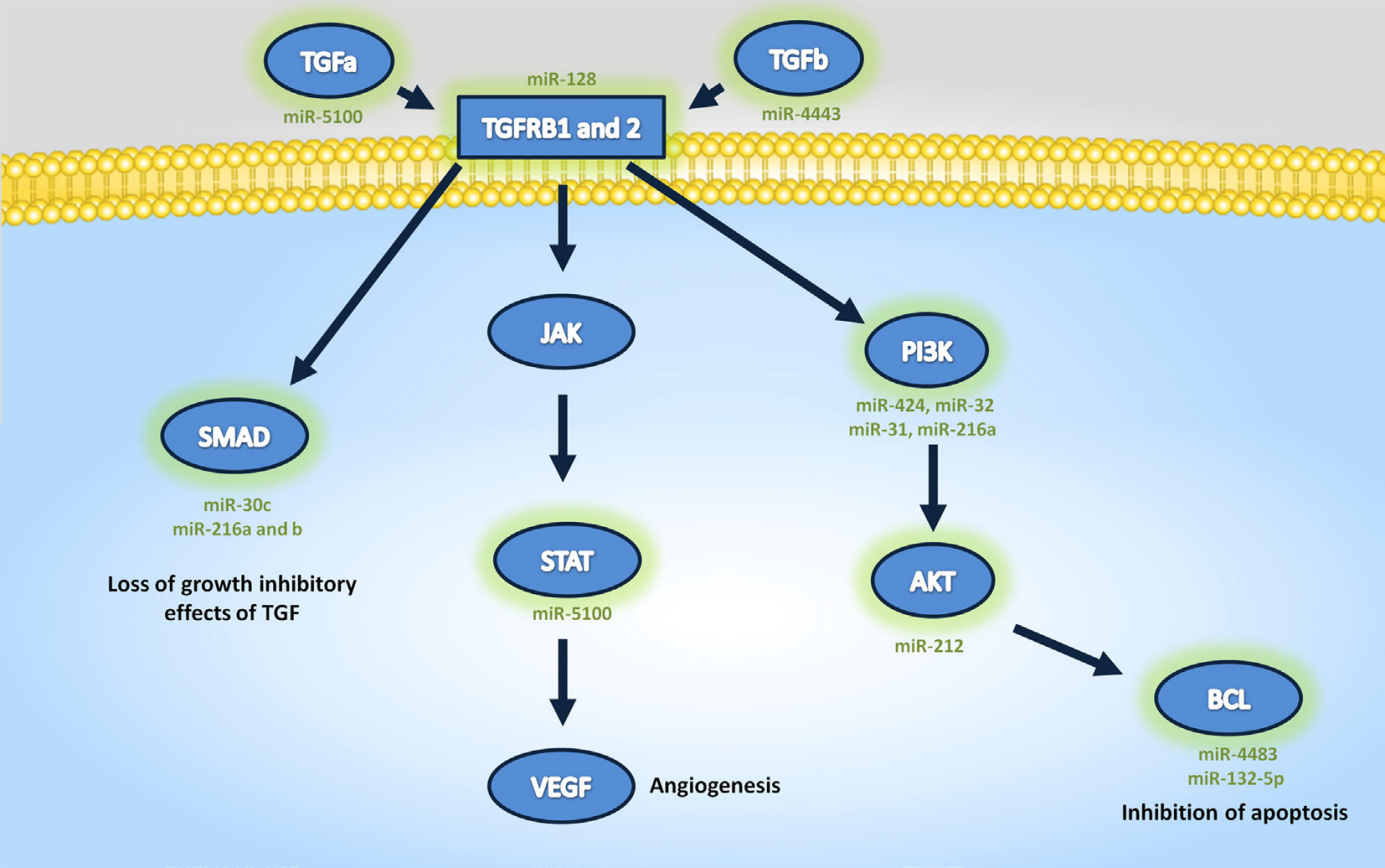
Summary of the main target genes targeted regulated microRNAs.

Tarbase did not contain interaction data for four of the differentially expressed miRNA that were identified, but analysis of the GO (Table 4) and pathway targets (Table 5) of the 12 miRNA identified reveal that these miRNA target genes are involved in key biological processes. In addition to several targeted cancer pathways, other cancer-related processes, such as focal adhesion, cell cycle, and critical signaling pathways were identified. Among the top regulated Kegg pathways were pathways in cancer (Figure S1 in Supplementary Material), proteoglycans in cancer (Figure S2 in Supplementary Material), TGF-β signaling pathways (Figure S3 in Supplementary Material), and FoxO signaling pathways (Figure S4 in Supplementary Material). A summary of the main target genes of the regulated miRNAs is provided in Figure 1. These data expose the potential ability of the serum from HNSCC patients to affect expression of genes in key cellular pathways by altering levels of regulating miRNAs.

**Table 4:** Gene ontology terms for biological processes of target genes of 16 microRNAs (miRNAs) differentially expressed in cells treated with serum from healthy individuals compared with head and neck squamous cell carcinomas (HNSCC) patients.

Gene ontology category-biological process	*P*-value	Genes^a^	miRNAs^b^
Response to stress	<3.33E−16	330	6
Catabolic process	<3.33E−16	305	7
Viral process	<3.33E−16	124	7
Symbiosis, encompassing mutualism through parasitism	<3.33E−16	138	7
Biological process	<3.33E−16	2,006	8
Biosynthetic process	<3.33E−16	665	8
Cellular nitrogen compound metabolic process	<3.33E−16	813	8
Cellular protein modification process	<3.33E−16	425	9
Gene expression	<3.33E−16	171	9
Mitotic cell cycle	3.33E−16	93	8
Neurotrophin trk receptor signaling pathway	2.67E−13	54	5
Cellular protein metabolic process	3.89E−12	80	5
Cellular component assembly	1.44E−11	184	5
MRNA metabolic process	6.96E−11	48	5
Small molecule metabolic process	2.99E−10	307	6
RNA metabolic process	8.23E−10	53	5
Membrane organization	4.12E−09	98	5
Fc-epsilon receptor signaling pathway	2.08E−08	35	5
Nucleobase-containing compound catabolic process	3.67E−08	128	5
Macromolecular complex assembly	2.67E−07	122	5
DNA metabolic process	3.81E−06	87	2
Epidermal growth factor receptor signaling pathway	5.96E−06	38	3
Transcription, DNA-templated	1.18E−05	286	3
Cell death	2.68E−05	128	4
Fibroblast growth factor receptor signaling pathway	5.45E−05	35	3
Activation of signaling protein activity involved in unfolded protein response	3.01E−04	17	4
Blood coagulation	8.64E−04	58	3
Viral life cycle	5.31E−03	14	2
Immune system process	7.60E−03	136	3
Protein complex assembly	8.01E−03	82	3
Endoplasmic reticulum unfolded protein response	8.49E−03	18	2
Cellular lipid metabolic process	1.69E−02	24	2
Termination of RNA polymerase to transcription	2.33E−02	10	2
TRIF-dependent toll-like receptor signaling pathway	2.91E−02	18	3

*^a^Number of genes in each process targeted by miRNAs*.

*^b^Number of miRNAs targeting genes in each process*.

**Table 5:** KEGG pathways of target genes of 16 microRNAs (miRNAs) differentially expressed in cells treated with serum from healthy individuals compared with head and neck squamous cell carcinomas patients.

KEGG pathway	*P*-value	Genes^a^	miRNAs^b^
Pathways in cancer	1.23E−02	95	11
PI3K-AKT signaling pathway	3.38E−02	82	12
HTLV-I infection	3.24E−02	68	12
Focal adhesion	2.35E−03	62	11
Proteoglycans in cancer	1.51E−06	60	12
Epstein–Barr virus infection	9.00E−03	58	12
Viral carcinogenesis	1.80E−03	56	11
Endocytosis	2.51E−02	52	11
FOXO signaling pathway	2.35E−03	46	11
Hepatitis B	3.62E−03	46	12
Protein processing in endoplasmic reticulum	2.51E−02	45	10
RNA transport	4.32E−02	44	11
Ubiquitin-mediated proteolysis	2.48E−02	41	10
Cell cycle	2.35E−03	40	10
Transcriptional misregulation in cancer	4.32E−02	40	10
Signaling pathways regulating pluripotency of stem cells	3.43E−02	39	11
Thyroid hormone signaling pathway	8.07E−03	37	11
Neurotrophin signaling pathway	1.38E−02	36	11
AMPK signaling pathway	4.32E−02	36	11
Oocyte meiosis	1.69E−04	35	10
Small cell lung cancer	4.01E−04	33	10
Choline metabolism in cancer	5.34E−03	33	10
Prostate cancer	2.35E−03	32	11
Adherens junction	7.74E−07	30	10
Estrogen signaling pathway	3.62E−03	30	11
Bacterial invasion of epithelial cells	9.64E−05	29	10
Erbb signaling pathway	1.62E−02	29	9
Chronic myeloid leukemia	4.96E−04	28	10
Progesterone-mediated oocyte maturation	3.28E−02	28	12
Salmonella infection	4.05E−02	27	11
Glioma	3.37E−06	26	9
Colorectal cancer	6.49E−05	26	11
Apoptosis	7.44E−03	24	9
P53 signaling pathway	9.00E−03	24	10
Prolactin signaling pathway	9.00E−03	24	10
Non-small cell lung cancer	1.69E−04	23	10
Shigellosis	2.35E−03	23	10
Pancreatic cancer	3.42E−03	23	9
Renal cell carcinoma	3.50E−02	22	8
Epithelial cell signaling in helicobacter pylori infection	4.62E−02	22	7
Melanoma	2.96E−02	21	8
Endometrial cancer	2.35E−03	20	11
Central carbon metabolism in cancer	3.24E−02	20	8
Synaptic vesicle cycle	3.38E−02	18	7
Bladder cancer	1.61E−02	15	7
Lysine degradation	3.38E−02	12	6
Thyroid cancer	5.23E−03	11	7
Fatty acid metabolism	5.16E−03	9	9

*^a^Number of genes in each pathway targeted by miRNAs*.

*^b^Number of miRNAs targeting genes in each pathway*.

### Other Critical Genes Are Affected by Exposure to Serum From Patients With HNSCC

**Figure 2:**
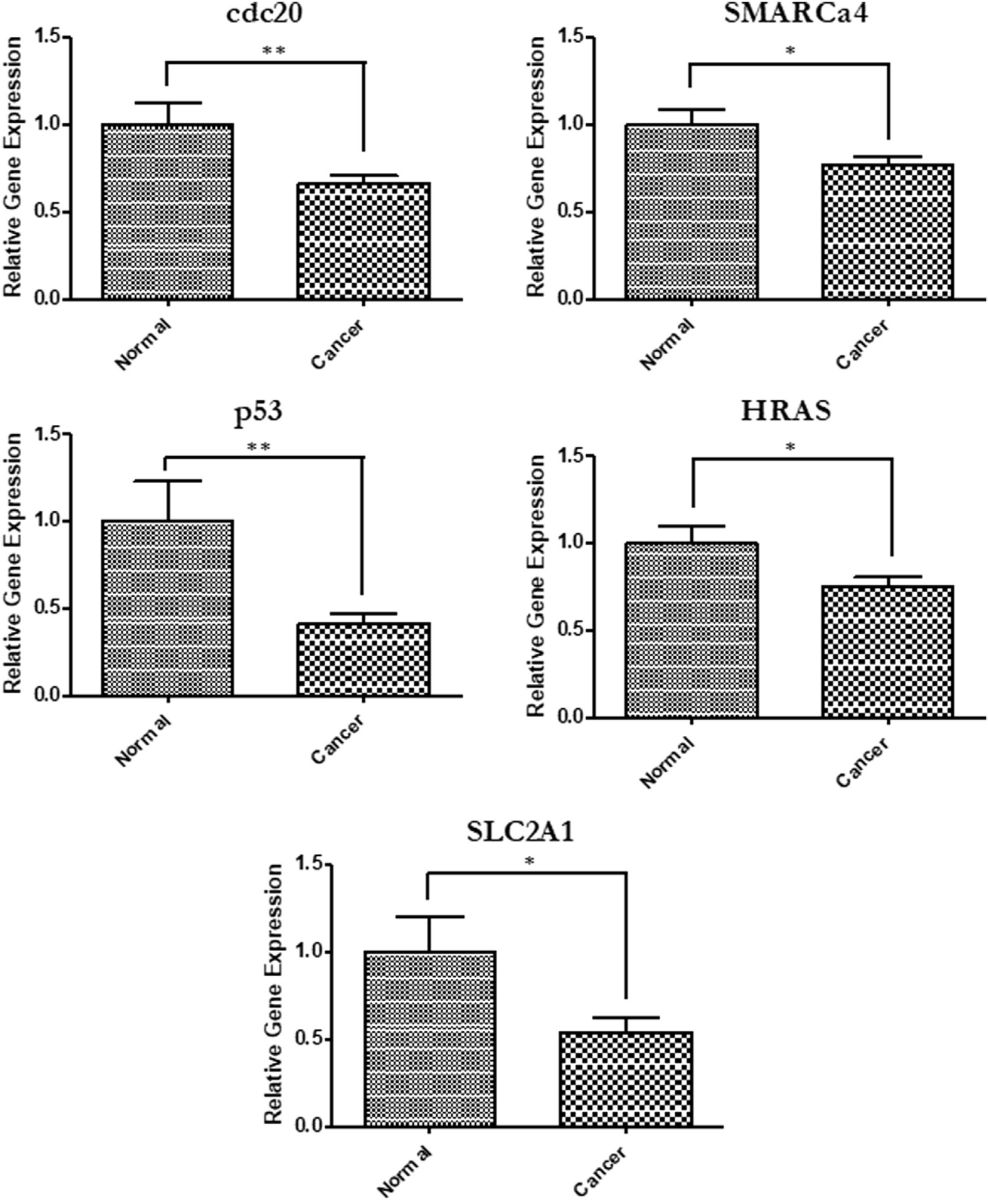
Gene expression changes in cells exposed to head and neck squamous cell carcinomas patient serum. Quantitative PCR was used to determine relative gene expression of several cancer-related genes. Primers used for each analysis are listed in Table 2. SLC2a1 (*p* = 0.0198), p53 (*p* = 0.0011), cdc20 (*p* = 0.0043), HRAS (*p* = 0.0306), and Smarca4 (*p* = 0.0235).

In order to relate the differentially expressed miRNA to gene expression, we performed quantitative PCR to measure mRNA levels of some critical genes involved in cancer and targeted by the differentially expressed miRNAs. A total of 14 genes were tested and 5 were found to have significantly reduced expressions (*p* < 0.05) in cells that were treated with serum from cancer patients compared with those treated with serum from healthy individuals (Figure 2). This further demonstrates the ability of HNSCC serum to alter the expression of cancer-related genes when introduced to cells.

## Discussion

The miRNAs identified in the cells treated with the serum of HNSCC patients in this study interact with target mRNAs known to be involved in oncogenic processes, including proliferation, survival, and angiogenesis, and many have been shown to be dysregulated in cancer. Dysregulation of proteins involved in the regulation of apoptosis can have oncogenic consequences, as evidenced by the many cell cycle regulating proteins that are either tumor suppressors or oncogenes (44). One of these proteins that is frequently mutated or dysregulated in cancer is p53 This protein acts as a guardian of the DNA damage cell cycle checkpoint and is responsible for initiating apoptosis when damage is repaired (44). The tumor suppressor gene, p53, plays a major role in a variety of cancers, including head and neck (45).

In this study, we identified MDM2 as a target of miR-32-5p, which we found to be downregulated in cells treated with HNSCC serum compared with those treated with serum from healthy donors. This interaction is supported by studies demonstrating the ability of miR-32 to cause accumulation of the tumor suppressor p53 by facilitating degradation of MDM2 (46). Importantly, our additional analysis of previously published studies of serum-circulating miRNA conducted in our laboratory (20)—as well as miRNA differentially regulated in cells treated with serum from HNSCC patients in this study—indicated that miR-32-5p represents the single common miRNA differentially regulated in the circulation of HNSCC patients, and also in cells treated with serum from HNSCC patients. Further, Sirt1, a target of differentially expressed miR-128-3p and miR-32-5p, also deacetylates p53, thereby inhibiting its transcriptional activity (47). Thus, reduced expression of these miRNA could facilitate p53 inhibition in the treated cells by increasing Sirt1 expression. Notably, we also found that exposure to serum from HNSCC patients resulted in decreased p53 expression.

Two other miRNAs found in this study to be downregulated by exposure to the HNSCC patient serum, namely miR-212-5p and miR-132-5p, also target proteins involved in cell cycle regulation. MiR-212-5p targets CCND1 and miR-132-5p targets Bcl2. The protein product of CCND1, cyclin D1, cooperates with other proteins and will facilitate cell cycle progression from G1 to S phase (48), while Bcl2 inhibits apoptosis by blocking the activity of pro-apoptotic proteins like Bax, Bak, and p53 (49). MiR-132 and miR-212 are formed by differential processing of the same primary miRNA. Upregulation of this gene cluster was shown to increase apoptosis and downregulate cyclin D1 and induce cell cycle arrest (50).

Mir-31 has been shown to have oncogenic influence (51) and the potential as a biomarker for detection and to monitor treatment response and prognosis (52–55). Along with miR-135b-5p, miR-31-3p has also been shown by our lab to be upregulated in tumor tissue of HNSCC patients compared with adjacent normal tissue (56). Both miR-31-3p and miR-135b-5p were found to be upregulated in cells treated with serum from cancer patients in this study.

Mir-128 has been shown to be downregulated in many types of cancer and acts as a tumor suppressor in HNSCC specifically. Overexpression of miR-128 in HNSCC cell lines inhibited cell growth and downregulated anti-apoptotic proteins, including MDM2, Bcl2, and NFkB (57). MCL1, another anti-apoptotic member of the BCL2 family, is a target of miR-32-5p, and overexpression of miR-32 was shown to induce apoptosis (58). MiR-135-5p, one of the miRNAs found to be upregulated in cells treated with serum from HNSCC patients, targets the tumor suppressor APC and promotes cell growth in colorectal cancer (59).

We found that SLC2A1, a target of miR-30c-2-3p, was downregulated in cells treated with cancer serum but not with normal serum. Although the other downregulated genes identified in this study were not identified as direct targets of the differentially expressed miRNA, they could be indirectly affected by other genes that are targeted. Several of the miRNAs identified target genes involved in regulating gene expression, such as transcription factors and histone modifiers, potentially enable them to pleiotropically alter gene expression in the treated cells.

In summary, 16 miRNAs were found to be differentially expressed in cells treated with serum from HNSCC patients compared with cells treated with serum from healthy humans. These miRNAs are involved in essential cellular processes that are dysregulated in cancer cells. Further, the serum altered the expression of several cancer-related genes. While other studies have demonstrated differences in miRNA expression between healthy and cancer tissue in HNSCC, as well as in the circulation of HNSCC patients, this study goes further to demonstrate the ability of serum from cancer patients to alter the expression of both genes and miRNAs in exposed cells. This concept could have a significant impact on the study of metastatic cancer as it reveals the ability of cancer-associated factors in circulation to affect the expression of genes and regulatory elements in distal cells in favor of tumorigenesis. This could also contribute to the systemic effects that cancer has on patient health. Further, better understanding of the role of serum associated factors on the regulation of tumorigenesis can lead to new therapeutic approaches. More importantly, these findings can help to develop new diagnostics using established tumor-specific cell lines or single-cell *in vitro* assays for personalized treatments and non-invasive follow-ups after surgical and oncological treatment.

## Ethics Statement

All methods in this study followed the protocol approved by the Institutional Review Board of Poznan University of Medical Sciences in Poznan, Poland. All experiments were performed in accordance with relevant guidelines and regulations. Informed consent for participation in the study has been obtained from all patients included in the study in accordance with the Declaration of Helsinki.

## Author Contributions

BA designed the study, performed experiments, and wrote the manuscript. AS analyzed the data, prepared the figures, and wrote the manuscript. BV performed experiments. YL analyzed the data, performed statistical analysis. MM designed experiments, prepared the manuscript. MS, JP, and EM gathered and provided patients data, reviewed the manuscript. WB gathered and provided patient data, processed the blood samples. WG obtained the tissue specimens during the surgical procedure. PG designed the study, obtained the tissue specimens during surgical procedures, selected the patients, and wrote the manuscript. MM designed the study, wrote the manuscript, and supervised the experiments.

## Conflict of Interest Statement

The authors declare that the research was conducted in the absence of any commercial or financial relationships that could be construed as a potential conflict of interest.

## Supplementary Material

Figure S1 KEGG pathway (40) analysis: pathways in cancer. Diagram of pathways in cancer genes and interactions. Genes highlighted in yellow are targeted by one of the differently expressed microRNA (miRNA) and genes highlighted in orange are targeted by more than one miRNA.

Figure S2 KEGG pathway (40) analysis: proteoglycans in cancer. Diagram of pathways in cancer genes and interactions. Genes highlighted in yellow are targeted by one of the differently expressed microRNA (miRNA) and genes highlighted in orange are targeted by more than one miRNA.

Figure S3 KEGG pathway (40) analysis: TGF-β signaling pathways. Diagram of pathways in cancer genes and interactions. Genes highlighted in yellow are targeted by one of the differently expressed microRNA (miRNA) and genes highlighted in orange are targeted by more than one miRNA.

Figure S4 KEGG pathway (40) analysis: FoxO signaling pathway. Diagram of pathways in cancer genes and interactions. Genes highlighted in yellow are targeted by one of the differently expressed microRNA (miRNA) and genes highlighted in orange are targeted by more than one miRNA.
