# Supplementary figures and images for: Blood Serum From Head and Neck Squamous Cell Carcinoma Patients Induces Altered MicroRNA and Target Gene Expression Profile in Treated Cells
Source: Front Oncol. 2018 Jun 11;8:217. doi: 10.3389/fonc.2018.00217 (PMC6004400; doi:10.3389/fonc.2018.00217)

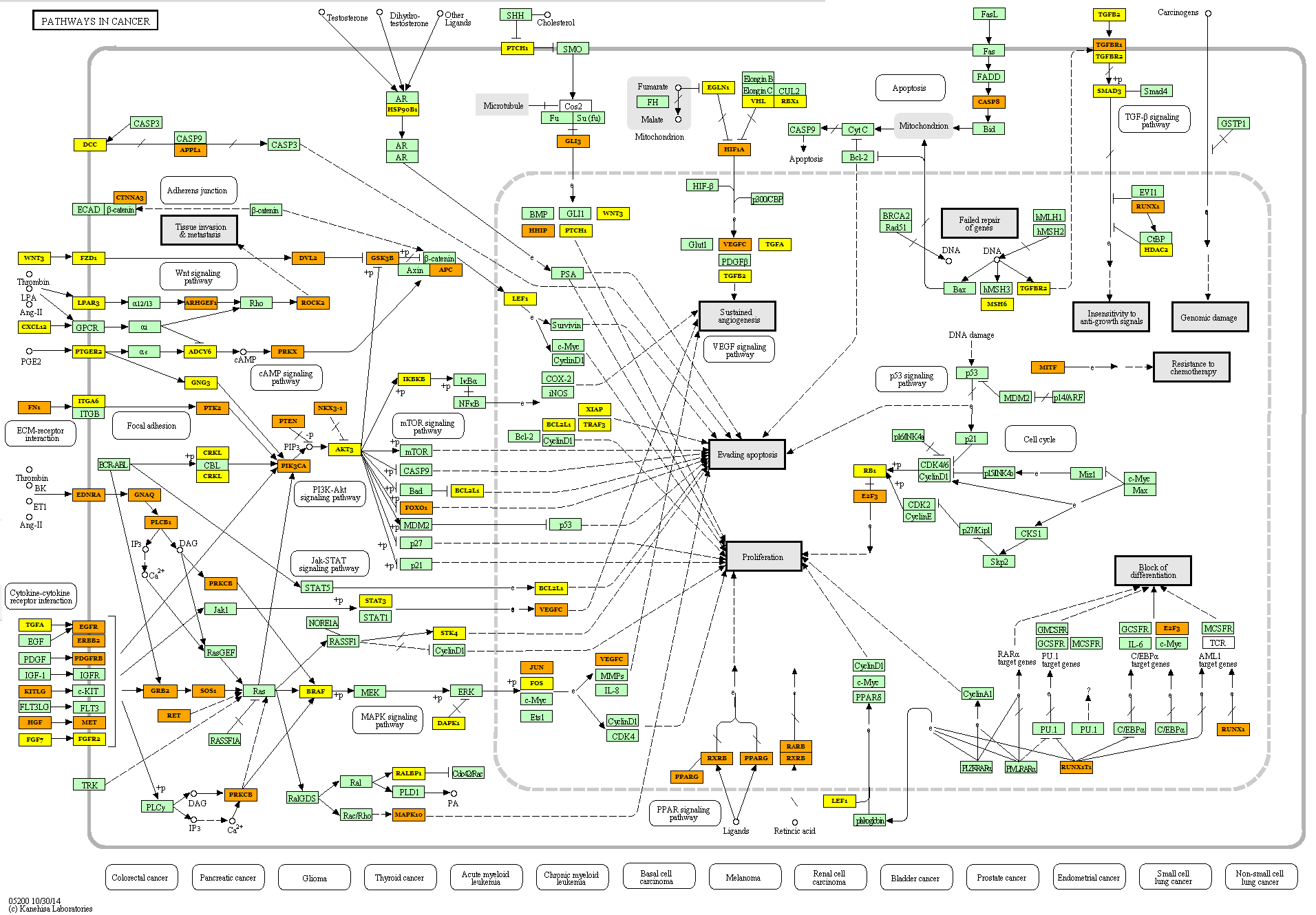

Supplement: Figure S1 — KEGG pathway (40) analysis: pathways in cancer. Diagram of pathways in cancer genes and interactions. Genes highlighted in yellow are targeted by one of the differently expressed microRNA (miRNA) and genes highlighted in orange are targeted by more than one miRNA. [file Image_1.tif]

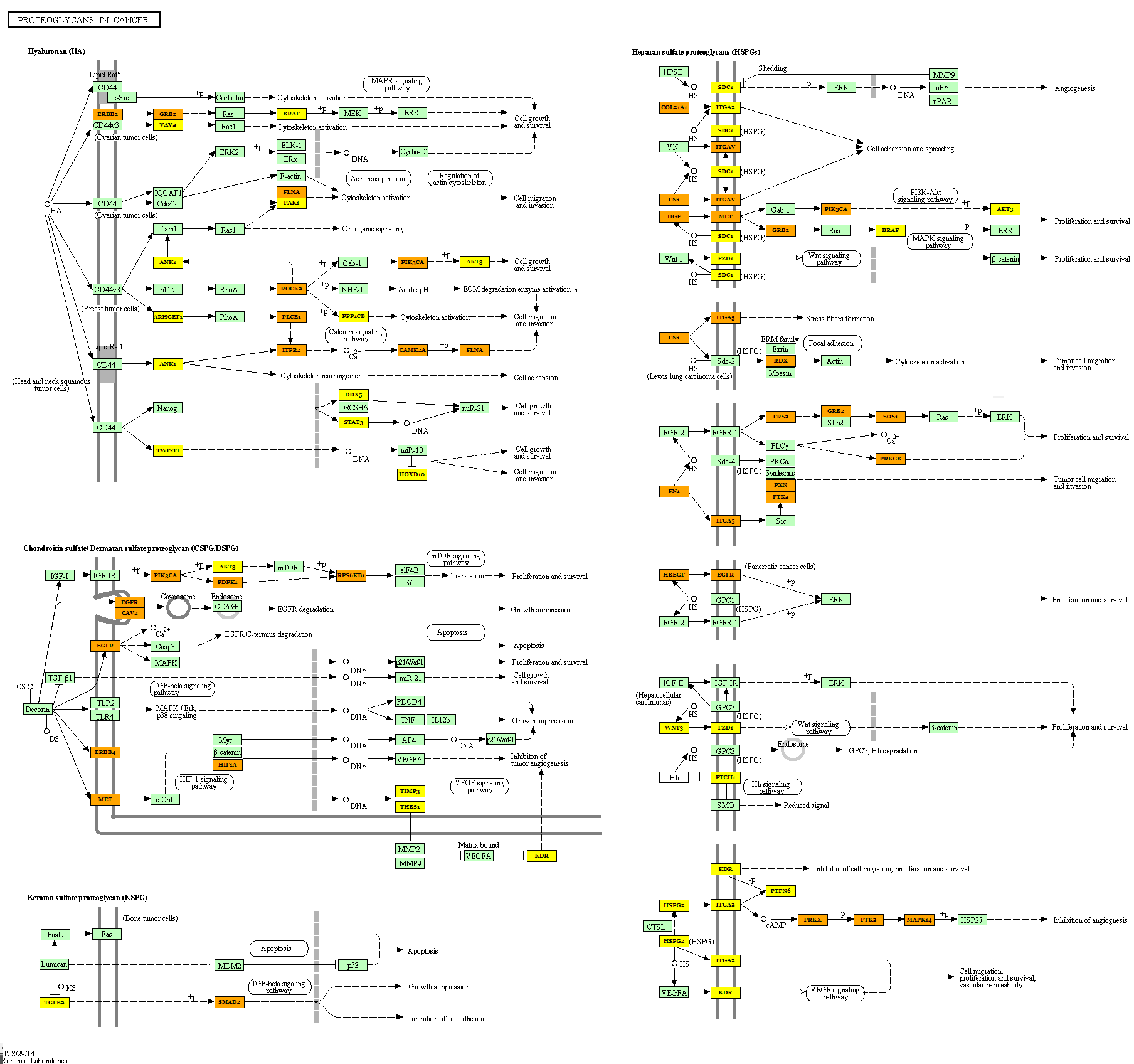

Supplement: Figure S2 — KEGG pathway (40) analysis: proteoglycans in cancer. Diagram of pathways in cancer genes and interactions. Genes highlighted in yellow are targeted by one of the differently expressed microRNA (miRNA) and genes highlighted in orange are targeted by more than one miRNA. [file Image_2.tif]

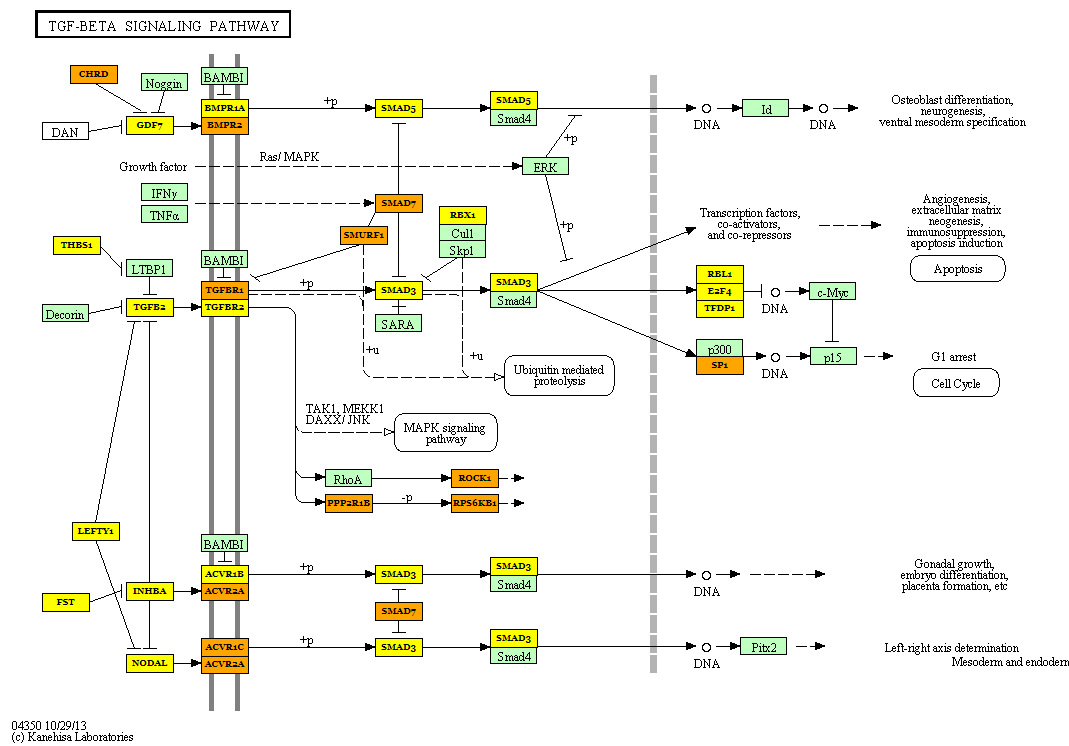

Supplement: Figure S3 — KEGG pathway (40) analysis: TGF-β signaling pathways. Diagram of pathways in cancer genes and interactions. Genes highlighted in yellow are targeted by one of the differently expressed microRNA (miRNA) and genes highlighted in orange are targeted by more than one miRNA. [file Image_3.tif]

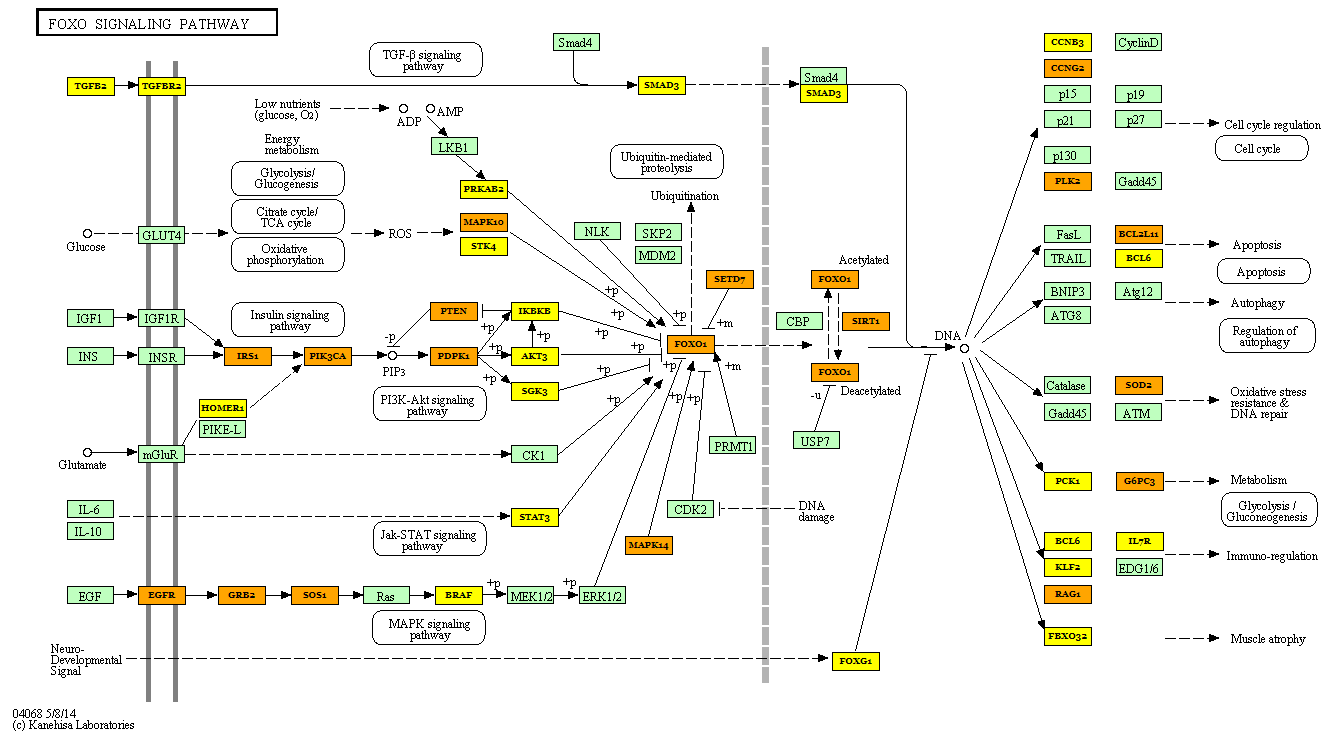

Supplement: Figure S4 — KEGG pathway (40) analysis: FoxO signaling pathway. Diagram of pathways in cancer genes and interactions. Genes highlighted in yellow are targeted by one of the differently expressed microRNA (miRNA) and genes highlighted in orange are targeted by more than one miRNA. [file Image_4.tif]
